# The effects of lycopene intake and exercise on bone health in young female rats

**DOI:** 10.1186/1550-2783-8-S1-P30

**Published:** 2011-11-07

**Authors:** Yuya Kakutani, Yuki Aikawa, Ikuko Ezawa, Naomi Omi

**Affiliations:** 1 Laboratory of Sports Nutrition, University of Tsukuba Graduate School of Comprehensive Human Sciences, Tsukuba 305-8574, Japan; 2 Japan Women’s University, Bunkyo 112-8681, Japan

## Background

Oxidative stress caused by free radicals and antioxidant imbalance damage cellular lipids, proteins and DNA. Recently, some studies have demonstrated that oxidative stress is a key modulator of bone cell function and that oxidative status influences the pathophysiology of bone. Endurance exercise is effective for antioxidant enzyme activity enhancement and the bone formation enhancement. On the other hand, lycopene is a kind of carotenoids had a higher antioxidant capability to reduce oxidative stress caused by exercise. In addition, several studies have reported that lycopene is effective for suppressing bone resorption. Thus, we considered that combining exercise and lycopene can contribute to bone health. The aim of this study was to investigate the effects of combining exercise and lycopene intake on bone health.

## Methods

Female Wistar rats, 6 weeks old, were fed for 10 weeks. Rats were divided into four groups for; sedentary control (C), sedentary control with lycopene intake (Ly), training exercise (T), and training with lycopene intake (TLy). Incidentally, concentration of lycopene in the diet was adjusted to 100ppm using a tomato oleoresin containing 6% lycopene. Rats in the two training groups were trained at 6 times a week for 9 weeks by treadmill running. All rats were given diets and distilled water *ad libitum*. Breaking force and breaking energy of femoral diaphysis and bone mineral content (BMC) and bone mineral density (BMD) of tibia were measured after dissection and were corrected body weight except for BMD. Data were analyzed using un-paired t test and two-way ANOVA with an alpha level of 0.05.

## Results

Breaking force, breaking energy, BMC and BMD in training groups (T and TLy) showed significant increases as compared with sedentary groups (C and Ly) (8.0 ± 0.17 vs. 9.2 ± 0.12 *10^6^ dyn/100g BW; 4.3 ± 0.19 vs. 5.4 ± 0.19 *10^6^ dyn/100g BW; 89.4 ± 0.67 vs. 101.9 ± 0.66 mg/100g BW; 123.6 ± 0.53 vs. 128.5 ± 0.63 mg/cm^2^; p < 0.001 respectively). Breaking force and breaking energy in lycopene diet groups (Ly and TLy) showed significant increases as compared with control diet (C and T) (8.2 ± 0.19 vs. 9.0 ± 0.14 *10^6^ dyn/100g BW; p < 0.01, 4.5 ± 0.20 vs. 5.2 ± 0.21 *10^6^ dyn/100g BW; p < 0.05), but not for BMC and BMD. Additionally, it was not found for lycopene intake and training exercise synergistic effect.

## Conclusions

In conclusion, these results suggest that lycopene intake exhibited positive effect on bone strength but not on BMD.

